# Estudio *in vitro* del efecto de tres solventes comerciales sobre conos de gutapercha utilizados para técnica en frío y termoplastificada

**DOI:** 10.21142/2523-2754-1002-2022-104

**Published:** 2022-06-27

**Authors:** Paul Josep Saavedra Gonzales, Miguel Angel Cabrera Iberico

**Affiliations:** 1 División de Carielogía y Endodoncia, Carrera de Odontología de la Universidad Científica del Sur. Lima, Perú. paul140217@gmail.com, mcabrera@cientifica.edu.pe Universidad Científica del Sur División de Carielogía y Endodoncia Carrera de Odontología Universidad Científica del Sur Lima Peru paul140217@gmail.com mcabrera@cientifica.edu.pe

**Keywords:** solvente, gutapercha, técnica en frío, técnica termoplastificada, solvent, gutta-percha, cold technique, thermoplastic technique

## Abstract

El objetivo de esta investigación fue comparar la acción solvente del óleo de naranja, eucaliptol y óleo experimental de hierba luisa sobre conos de gutapercha para técnica en frío y técnica termoplastificada.

**Metodología:**

La muestra estuvo compuesta por 80 conos de gutapercha n.^o^ 80 Endomedic y F3 ProTaper Universal. Los conos se dividieron en 4 grupos (n = 20) según el tipo de solvente y, a la vez, cada uno se dividió en dos subgrupos (n = 10) según el tiempo de exposición (5 min y 10 min). Los conos se sumergieron en el solvente respectivo durante 5 o 10 minutos. Luego, se lavaron con 5 ml de alcohol durante 5 minutos y se enjuagaron con agua. Finalmente, luego de secar a temperatura ambiente por una hora, la acción solvente se registró en gramos (g) de pérdida de peso. Los resultados fueron analizados y comparados mediante la prueba de Tukey (p < 0,05).

**Resultados:**

El cono Endomedic expuesto durante 5 min a eucaliptol obtuvo el promedio de peso más bajo (0,0625 ± 0,0009 g), seguido del óleo de hierba luisa (0,0629 ± 0,0003 g) y óleo de naranja (0,0629 ± 0,0010 g), aunque no se encontró diferencia significativa (p > 0,05) entre cada uno.

**Conclusión:**

El solvente experimental de hierba luisa, el eucaliptol y el óleo de naranja presentaron un efecto solvente similar sobre los conos de gutarpercha para técnica en frío y termoplastificada. A nivel clínico los tres solventes estudiados podrían usarse en el retratamiento endodóntico como soluciones alternativas de similar efecto solvente.

## Abstract (en)

The objective of this research was to compare the solvent action of orange oil, eucalyptol and experimental lemongrass oil on gutta-percha cones for cold technique and thermoplastic technique.

**Methodology:**

The sample consisted of 80 gutta-percha cones No. 80 Endomedic and F3 ProTaper Universal. The cones were divided into 4 groups (n = 20) according to the type of solvent, and each one was divided into two subgroups (n = 10) according to the exposure time (5 min and 10 min). The cones were immersed in the respective solvent for 5 or 10 minutes. Then, they were washed with 5 ml of alcohol for 5 minutes and rinsed with water. Finally, after drying at room temperature for one hour, the solvent action was recorded in grams (g) of weight loss. The results were analyzed and compared using Tukey's test (p<0.05).

**Results:**

The Endomedic cone exposed for 5 min to eucalyptol obtained the lowest average weight (0.0625 ± 0.0009 g), followed by Lemongrass oil (0.0629 ± 0.0003 g) and orange oil (0.0629 ± 0.0010 g), although not a significant difference (p > 0.05) was found between each one.

**Conclusion:**

The experimental solvent of Lemongrass, eucalyptol and orange oil presented a similar solvent effect on the guta-percha cone for cold and thermoplastic technique. At a clinical level, the three solvents studied could be used in endodontic retreatment as alternative solutions with a similar solvent effect.

## INTRODUCCIÓN

La principal causa del "fracaso" de las endodoncias es la persistencia de infecciones microbianas intra y extraradiculares. Además, durante el retratamiento de endodoncia, para la remoción de materiales de relleno, existen varias técnicas que incluyen limas manuales, limas rotatorias e instrumentos ultrasónicos solos o en combinación con el calor, o con el uso de algún solvente. La gutapercha y los cementos endodónticos, son los materiales de obturación más utilizados. Por ello, su eliminación es un requisito previo para cualquier retratamiento ^1^^-^^3^.

Por lo general, la remoción de gutapercha no requiere un gran esfuerzo al usar las técnicas mencionadas. Sin embargo, los estudios postulan que los residuos de material de relleno, especialmente los selladores, están presentes en las paredes del canal y en sus ramificaciones microscópicas y pueden permanecer inaccesibles o resistentes a la disolución ^4^^,^^5^. En muchos casos, la eliminación completa de la gutapercha es variable. Los solventes orgánicos cumplen un papel importante en la eliminación de gutapercha. Esto facilita la buena irrigación y desinfección eficiente del conducto radicular y, por lo tanto, la probabilidad de una instrumentación y eliminación de residuos contaminados fuera del conducto radicular, mejorando así el pronóstico a largo plazo. Asimismo, los aceites esenciales como el aceite de naranja, el aceite de eucalipto y el aceite de pino, mientras que otros son capaces de disolver gutapercha. Se ha informado de su utilidad, seguridad, biocompatibilidad y bajo efecto carcinogénico ^6^^,^^7^.

La destilación por arrastre de vapor es uno de los medios que facilita la extracción del aceite de naranja (*Citrus aurantium*). Este aceite esencial se utiliza para reblandecer la gutapercha, sobre todo en piezas obturadas con cemento a base de óxido de zinc-eugenol. Por excelencia el aceite de naranja es un solvente de elección comparado con otros solventes inorgánicos y potencialmente tóxicos ^8^^,^^9^.

*Cymbopogon citratus* es una planta aromática de la familia Gramineae conocida como hierba luisa o hierba de limón. El olor a limón podría atribuirse a la existencia de un monoterpeno cíclico (citral). Sin embargo, también posee un grupo considerable de cetonas, alcoholes, fenoles, terpenos, flavonoides, saponinas, esteroides, taninos, alcaloides, geraniales, terpernoides, polifenoles, ésteres, aldehídos, ácidos grasos y otros constituyentes fitoquímicos que posee actividades farmacológicas ^10^.

Adicionalmente, el aceite esencial de hierba luisa posee propiedades farmacológicas tales como antiamebiano, antibacteriano (activo contra *Bacillus subtilis*, *E. coli*, Staphylococus aureus, *Salmonella paratyphi* y *Shigella flexneri*), antifúngico, antipalúdico, antinociceptivo, antiprotozoario, larvicida, anticonvulsivante, antioxidante y antiinflamatorio.^11^^,^^12^ Estudios clínicos hechos en esta especie, evaluaron las propiedades terapéuticas del mirceno, el cual posiblemente actúa como analgésico específico para algunas partes del cuerpo humano, su uso es tanto oral como tópico, y el citral es uno de los componentes que encontramos en mayor porcentaje ^13^.

El potencial genotóxico y citotóxico de los solventes cloroformo y xilol es conocido ^14^. Esto permitió la búsqueda de un material biocompatible que permita una eliminación eficaz del material de relleno, lo que da como resultado un tratamiento más rápido y una mejor capacidad de limpieza y desinfección. Aunque el xilol presente un alto rendimiento de disolución de gutapercha, presenta un efecto indeseable, por ejemplo, no puede ser inhalado por riesgo a presentar convulsiones, insomnio, excitación, y es muy irritante sobre las mucosas. Su uso debe ser cauteloso, ya que pueden llegar fácilmente a los tejidos periapicales y afectar el comportamiento de las células óseas, lo que altera el metabolismo óseo local y dificulta el proceso de curación ^15^^-^^18^. El eucaliptol se obtiene del aceite esencial de Eucalipto, es antiséptico y antibacteriano, y no causa irritación sobre las mucosas ^19^. Su principal inconveniente es el retraso en solubilizar la gutapercha, un hecho que puede ser mejorado si se la calienta ^20^. Generalmente, se han utilizado el cloroformo y xileno; sin embargo, las preocupaciones con respecto a su toxicidad exigen alternativas. Por lo tanto, el propósito de esta investigación fue comparar la acción solvente del óleo de naranja, el eucaliptol y el óleo experimental de hierba luisa sobre los conos de gutapercha para las técnicas en frío y termoplastificada. 

## MATERIALES Y MÉTODOS

Se realizó un estudio de tipo experimental *in vitro*. La muestra estuvo conformada por 80 conos de gutapercha y cada uno estuvo dentro de un recipiente de vidrio para una mejor administración de los solventes. Posteriormente, la muestra se dividió en 4 grupos experimentales (n = 20) según el tipo de solvente a utilizar. Así mismo, dentro de cada grupo los conos de gutapercha se dividieron en dos subgrupos (n = 10), el primer subgrupo fue expuesto durante 5 minutos al solvente de prueba, mientras que el segundo subgrupo se expuso durante 10 minutos, estos se seleccionaron siguiendo las recomendaciones del trabajo publicado previamente ^1^^,^^2^. Inicialmente los conos de gutapercha n.^o^ 80 Endomedic y conos de gutapercha F3 ProTaper universal se pesaron en una balanza digital analítica estándar de precisión (Boeco, Alemania) con capacidad máxima de 110 g, resolución 0,0001 g. Plataforma de 85 mm diámetro, tiempo de estabilización 3,5 segundos y calibración interna (automática). Luego, cada cono se colocó individualmente en placas de Petri de vidrio completamente sumergidas en 5 mL del solvente respectivo, a temperatura ambiente. Se controló el tiempo de inmersión con un cronometro; luego, el solvente se eliminó completamente fuera de la placa con la ayuda de una jeringa de plástico desechable. Posteriormente, se vertió alcohol etílico (por las características de miscibilidad, similitud molecular, polaridad y el principio de disolución) sobre el cono de gutapercha en la placa durante 5 minutos para el lavado inicial. Seguidamente, los conos se enjuagaron con 5 mL de agua destilada con ayuda de una jeringa durante 5 minutos. El agua destilada se retiró y el cono se secó en un papel absorbente a temperatura ambiente durante 1 hora. Después de estos procedimientos, los conos se pesaron nuevamente y el resultado de la acción solvente se obtuvo en base a la diferencia de pesos inicial y final. 

Para el análisis de los datos Se utilizó el *software* IBM SPSS Statistics 23, los valores se expresaron como media y desviación estándar (DE). Para el análisis estadístico, inicialmente, se evaluó la normalidad de los datos mediante la prueba de Kolmogorov-Smirnov, luego se utilizó la prueba de ANOVA de una vía o dos vías según corresponda, seguido de una prueba *post hoc* de Tukey. Se consideró estadísticamente significativo un valor de p < 0,05.

## RESULTADOS

La Tabla 1 muestra que el cono Endomedic expuesto durante 5 minutos a eucaliptol obtuvo el promedio de peso más bajo (0,0625 ± 0,0009 g), seguido de los conos expuestos al solvente de hierba luisa (0,0629 ± 0,0003 g) y óleo de naranja (0,0629 ± 0,0010 g), respectivamente. Luego de realizar la comparación múltiple mediante la prueba de Tukey y al evaluar la significancia estadística se evidenció según la Tabla 2 que no se encontró diferencia estadísticamente significativa entre los tres solventes evaluados en el presente trabajo de investigación (p > 0,05).

**Tabla 1:** Pesos (g) de los conos de gutapercha a los 5 minutos y 10 minutos según tipo de solvente y marca de cono

Grupos	Cono	Tiempo	n	Promedio	D. E.	Mínimo	Máximo
Óleo naranja	Endomedic	5 minutos	10	0,0629	0,0010	0,0610	0,0650
	ProTaper	10 minutos	10	0,0855	0,0008	0,0841	0,0865
Eucaliptol	Endomedic	5 minutos	10	0,0625	0,0009	0,0602	0,0632
	ProTaper	10 minutos	10	0,0852	0,0007	0,0843	0,0867
Hierba luisa	Endomedic	5 minutos	10	0,0629	0,0003	0,626	0,0637
	ProTaper	10 minutos	10	0,0858	0,0007	0,0842	0,0867

D. E. (Desviación estándar)

**Tabla 2:** Comparación de los valores del peso de los conos de gutapercha expuestos a los solventes a los 5 minutos

Grupos		5 min (p valor)	10 min (p valor)
Óleo naranja	Eucaliptol	1,000	1,000
	Hierba luisa	1,000	1,000
Eucaliptol	Óleo naranja	1,000	1,000
	Hierba luisa	0,999	1,000
Hierba luisa	Óleo naranja	1,000	1,000
	Eucaliptol	0,999	1,000

Prueba de ANOVA de una vía

## DISCUSIÓN

La clave para un retratamiento endodóntico exitoso es desbridar completamente el sistema de conductos del tejido pulpar infectado o necrótico. Así mismo, eliminar de manera eficiente los materiales de obturación del sistema de conductos radiculares. En tal sentido, se ha demostrado que los disolventes orgánicos ayudan a eliminar los materiales de obturación del conducto radicular; sin embargo, además de su efecto disolvente posee también efectos adversos para la salud ^18^. En tal sentido, es importante el estudio de nuevas fuentes de solventes que permitan obtener este efecto beneficioso con un menor riesgo.

Los aceites esenciales de naranja y eucaliptol estudiados por Limongi *et al*. (2003) ^20^ y Scelza *et al*. (2008) ^21^ reportaron que tenían la capacidad solvente para producir la limpieza del conducto radicular. El eucaliptol, incluso, reportó tener la capacidad de disolución de gutapercha y ser una alternativa eficiente además de no presentar efectos tóxicos ^9^^,^^22^^,^^23^. Por otro lado, el aceite de naranja es utilizado debido a que carece de efectos secundarios por lo descrito en la literatura científica y, a su vez, tiene una buena capacidad de disolución de gutapercha ^15^^-^^17^ de forma similar al presente estudio, en el que se puede concluir que el eucaliptol, el óleo experimental de hierba luisa y el óleo de naranja actúan de manera efectiva en la acción solvente de conos de gutapercha a los 5 y 10 minutos, y que el efecto de disolución de la gutapercha es dependiente del tiempo de exposición al solvente.

Se ha observado en base al estudio de Villavicencio *et al*. ^24^, que el eucaliptol genera en las gutaperchas una pérdida de peso de 0,00145 g a los 5 minutos y de 0,00481 g a los 10 minutos, por lo que se concluye que este tendría una mayor acción solvente en la disolución de la gutapercha que el óleo de naranja. Estos resultados contrastan con lo reportado en el presente trabajo de investigación, puesto que se observó que tanto el óleo de naranja como el eucaliptol no presentan diferencias significativas de pesos a los 5 y 10 minutos; sin embargo, un factor importante a tener en cuenta podría ser la marca del cono o la marca y procedencia de los solventes comerciales empleados. En el presente estudio se encontró que el óleo de naranja y el eucaliptol no presentaron diferencias significativas en cuanto a acción solvente sobre los conos de gutapercha en los tiempos de cinco y diez minutos, a diferencia de Martos *et al*. ^7^ que refieren que el xylol y el óleo de naranja presentaron mejores resultados sobre el eucaliptol, aunque en el estudio que realizaron se utilizaron tiempos de 2 y 10 minutos de exposición. Los solventes comúnmente utilizados tienen un nivel de toxicidad como lo encontrado con el xylol; debido a que el solvente experimental de hierba luisa se convierte en un medio de posible uso en endodoncia, por lo que se requiere investigar concentraciones de uso en relación a pruebas de biocompatibilidad en futuros estudios.

Finalmente, respecto al óleo experimental de hierba luisa, se ha demostrado que podría ser una alternativa en los retratamientos por presentar una acción de disolución de gutapercha a partir de los diez minutos, por lo cual se podría iniciar una línea de investigación utilizando distintos métodos de prueba, tiempos, tipos y grosores de gutapercha, con la finalidad de buscar alternativas adecuadas al mejor uso en la práctica clínica en endodoncia.

## CONCLUSIONES

El solvente experimental de hierba luisa presentó similar efecto solvente sobre el cono de gutarpercha para técnica en frío y termoplastificada que el uso del eucaliptol y óleo de naranja. A nivel clínico, los tres solventes estudiados podrían usarse en el retratamiento endodóntico como soluciones alternativas de similar efecto solvente. Se requieren investigaciones futuras y más robustas sobre el poder solvente de la hierba luisa, debido a que podría ser una alternativa en la utilización de retratamientos en endodoncia.

## Fuente de financiamiento:

Autofinanciado
